# Supplementary figures and images for: Comparison of glenohumeral joint kinematics between swimmers clinically classified with multidirectional instability and asymptomatic controls
Source: PLoS One. 2025 Oct 28;20(10):e0335045. doi: 10.1371/journal.pone.0335045 (PMC12561955; doi:10.1371/journal.pone.0335045)

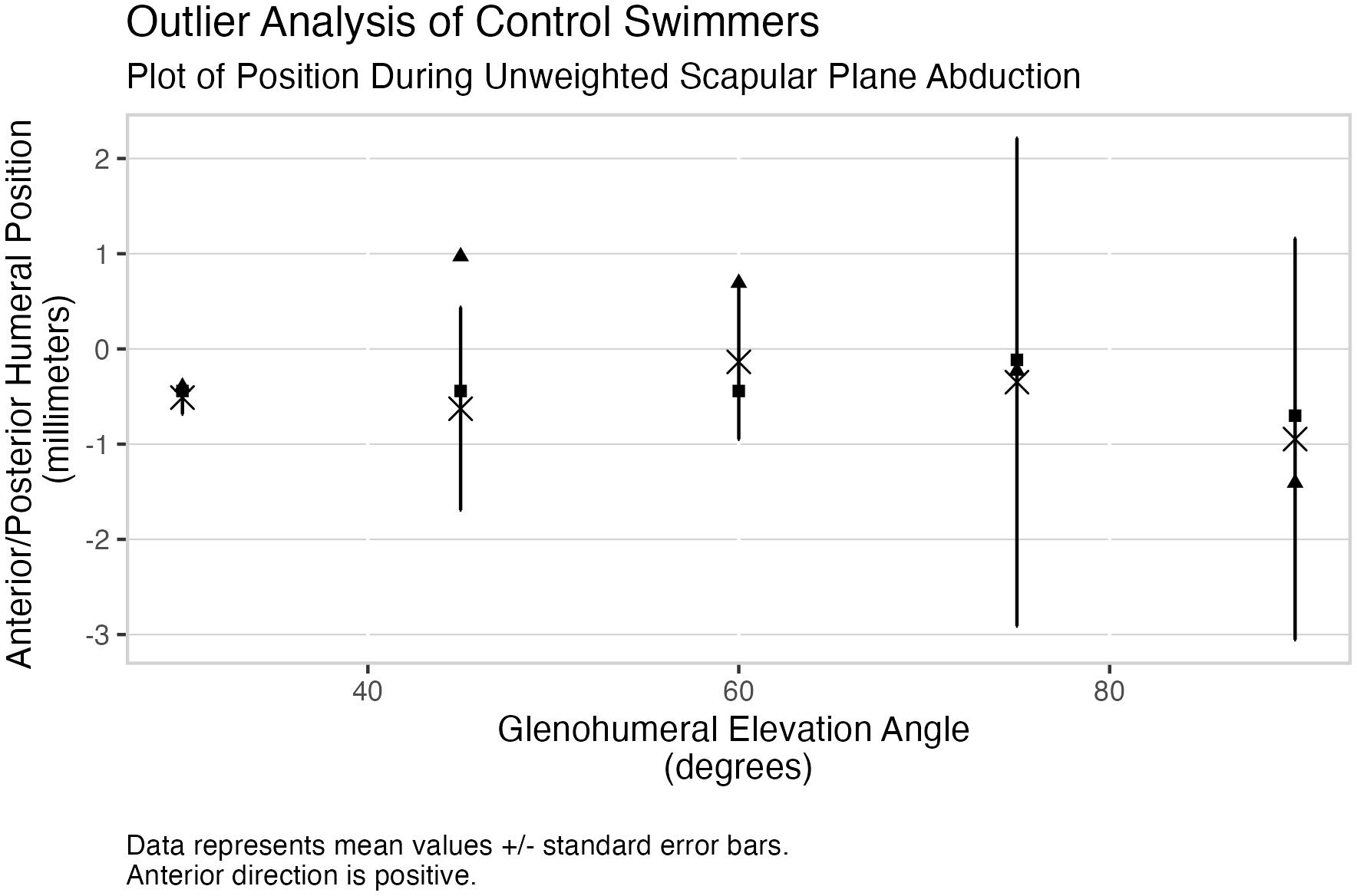

Supplement: S1 Fig 4 — Caption: triangles and squares represent individual data from the two competitive swimmers included in the control group compared to the IQR (vertical black lines) of the control group and control group means, indicated by the “X”. (TIF) [file pone.0335045.s002.tif]

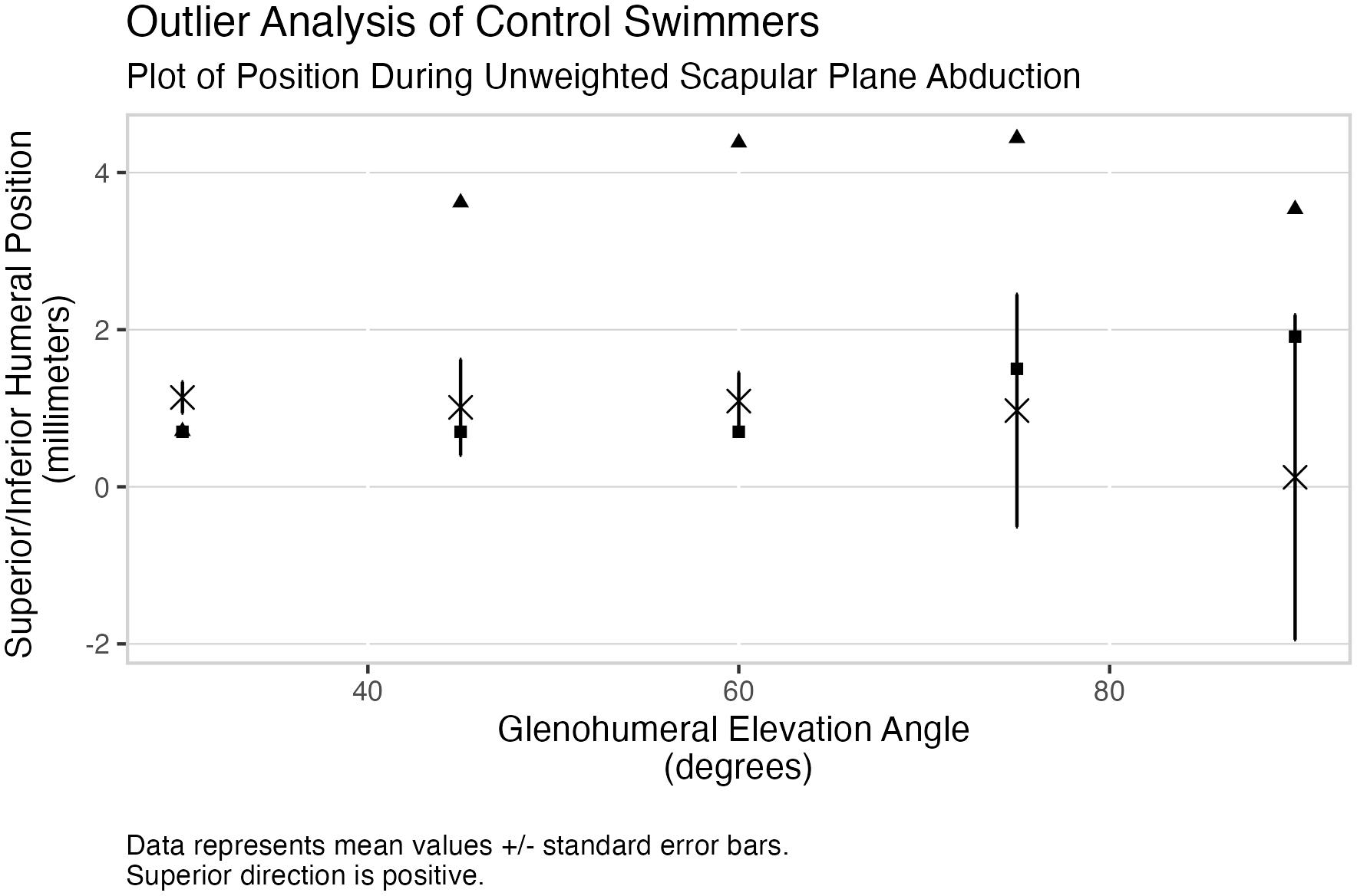

Supplement: S2 Fig 5 — Caption: triangles and squares represent individual data from the two competitive swimmers included in the control group compared to the IQR (vertical black lines) of the control group and control group means, indicated by the “X”. (TIF) [file pone.0335045.s003.tif]
