# Supplementary material for: Comparison of glenohumeral joint kinematics between swimmers clinically classified with multidirectional instability and asymptomatic controls
Source: PLoS One. 2025 Oct 28;20(10):e0335045. doi: 10.1371/journal.pone.0335045 (PMC12561955; doi:10.1371/journal.pone.0335045)
Supplement: Attachment — Submitted filename: Reviewer Notes PLOS.pdf [file pone.0335045.s005.pdf]

Reviewer Feedback Letter

Dear Authors,

Dear Authors,

Thank you for the opportunity to review your manuscript, *“Comparison of glenohumeral joint kinematics between swimmers clinically classified with multidirectional instability and asymptomatic controls.”* The authors make an important contribution to the effort of identifying novel and more objective ways to classify multidirectional instability (MDI) in overhead athletes compared to asymptomatic controls. The paper is well organized, clearly written, and provides helpful considerations that will help pave the way for future studies using kinematic variables to detect MDI. Please see my review comments below.

Reviewer Comments

1. **Recruitment timelines (Lines 104–106 & Lines 118–119)**
   - Can you specify timelines for recruitment of the clinical instability group as part of the larger study compared to when controls were recruited?
   - (lines 118–119) state: “Participants for the clinical instability group were enrolled in a larger, ongoing research program investigating shoulder biomechanics and instability in competitive swimmers.” This does not seem to match the statement in lines 104–106: “Participants were recruited through outreach to local swimming communities using digital and physical announcements, including targeted email campaigns, community flyers, and social media communications. Recruitment started on 01 September 2021 and ended on 28 February 2023.”
2. **Power Analysis (Line 111)**
   - Recommend adding how power analysis was conducted, e.g., which program/method did you use? (e.g., G*Power, R “pwr” package, etc.).
3. **Recruitment rationale (Line 139)**
   - It is unclear why clinical tests were used to classify MDI and then compared with imaging outcomes to see if differences in kinematic variables were observed, rather than using another method of imaging as a more direct reference standard. This approach risks creating a “comparison of a comparison,” since prior studies have already contrasted clinical classification with dynamic imaging. Please clarify the rationale for this framework and how it advances beyond existing work.
4. **Control group description (Line 164)**
   - Here you state: “Competitive swimmers without a clinical classification of MDI and no shoulder pain were allowed to participate in the control group.”
   - This language is slightly confusing, as earlier you state that a clinical classification of MDI is present when the participant has MDI + pain, which makes it seem like a

participant could be classified as having clinical MDI even if there is no shoulder pain present?

5. **Rationale for IR/ER measurements (Lines 173–179)**
    - Can you provide a further explanation of why IR/ER was included as a clinical characteristic? More specifically, can you expand on what you mean by: “These goniometric measures were used for clinically relevant group descriptions”?
6. **MR vs. CT reconstruction (Lines 200–202)**
    - Can you provide a further explanation of why different methods of reconstruction were used for clinical instability (CT) and control groups (MR)?
7. **Explain why active unweighted scapular plane abduction (SAB) was chosen (Methods)**
    - I may have missed this, but it wasn’t clear to me why you chose SAB as the evaluated movement.
    - Can you please provide further justification of this movement in relation to your assessment procedure?
8. **Control group participants (Lines 294–295)**
    - Lines 294–295 state: “Two participants in the control group were competitive swimmers without MDI.”
    - This statement seems to contradict the earlier inclusion/exclusion criteria for the control group. Does this mean 8/10 had asymptomatic MDI or were not competitive swimmers?
9. **Demographics Table (Line 300)**
    - Can you provide a further explanation of why different methods of reconstruction were used for clinical instability (CT) and control groups (MR)?
10. **Table 1. BMI MDI Group (Line 300)**
    - Possible typo: upper range reported for BMI of 8.32 for clinical instability group.
11. **Table 2. Apprehension Sign Control (Line 302)**
    - Check 0/10 (0%) negative value in table. Would expect most controls to have a negative apprehension sign.
12. **Statistical vs. Clinical Significance (Lines 311–313)**
    - You report: “a main effect of group was observed, with the clinical instability group being significantly positioned anteriorly.”

- As phrased, “significantly positioned” risks being misinterpreted as clinically significant, when your MCID was set at 1.0 mm and the observed difference (0.8 mm) was below both this threshold and the measurement error (<0.9 mm).
- Recommend rewording to emphasize statistical significance only (e.g., “a statistically significant anterior shift was detected”).

13. **Discussion summary (Lines 321–322 vs. 409–410)**
    - Line 409 states: “During the first phase of elevation, humeral positional dispersion was significantly greater than in the second half (+17.40%)…”
    - This seems to contradict what is written in lines 321–322: “During the first 50% of motion, there was significantly less humeral positional dispersion (df = 1, 1, F = 23.04, P < 0.01).”
    - Can you please clarify this difference?
14. **Comparison to Overhead Athlete Literature (Lines 346–351)**
    - You write: “While others suggest that competitive overhead athletes typically exhibit increased ER and decreased IR [49], our results show that asymptomatic controls demonstrated greater ER and IR values.”
    - The cited reference (Wilk et al.) compares throwing vs. non-throwing arms, not pathological vs. healthy controls.
    - Consider clarifying this or citing studies that more directly compare symptomatic and asymptomatic athletes.
15. **Discussion summary (Lines 426–430)**
    - Although it is interesting that no significant changes between CPL were found between clinical MDI and control groups, I am not sure you can make the conclusion stated in lines 428–430: that the “data would support this concept with minimal difference in CPL between shoulders determined to be ‘unstable’ with clinical laxity testing compared to ‘stable’ control shoulders.”
    - As kinematic variables were tested in the present study using active shoulder abduction whereas clinical laxity testing was performed using manual maneuvers by an assessor passively loading the shoulder, which are entirely different, can you please clarify this difference?
16. **Swimmer Enrollment and Copers (Line 458)**
    - You state: “A potential way to address this in future research would be to enroll competitive swimmers in both groups.”

- Your Methods already specify: “Competitive swimmers without a clinical classification of MDI and no shoulder pain were allowed to participate in the control group.”
- Recommend revising this section to avoid suggesting swimmers were not already included in both groups, or clarify if this was not the case.
