# Supplementary material for: Comparison of glenohumeral joint kinematics between swimmers clinically classified with multidirectional instability and asymptomatic controls
Source: PLoS One. 2025 Oct 28;20(10):e0335045. doi: 10.1371/journal.pone.0335045 (PMC12561955; doi:10.1371/journal.pone.0335045)
Supplement: Attachment — Submitted filename: Response to Reviewers.docx [file pone.0335045.s006.docx]

Dear Dr. Srivastav,

Thank you for taking the time to review and manage our manuscript submission to PLOS ONE. As a team of authors, we have revised our manuscript according to the requirements of the Journal and reviewer comments. Our revised submission is stronger after making these changes.

Below you will find our responses (**bolded**) to each requirement/comment shared in the decision letter. The updated line numbers referenced in our responses relate to the un-tracked version of our revised submission. Once again, thank you for taking the time to review our manuscript and manage our submission.

Best,

Oliver Silverson, PhD

Submitting author

Journal Requirements:

| Journal Requirement | Please ensure that your manuscript meets PLOS ONE's style requirements, including those for file naming. |
| --- | --- |
| **Response** | **Thank you for providing links to the PLOS One style template and title page example. We have updated our revised manuscript according to the style templates provided. Our changes to the section headings and in-text citations are not tracked due to complexity of tracking changes to section headers and references.**  **All below described edits to the manuscript style have been tracked:**   - **We have revised our title page to conform with the requirements specified in the formatted sample title page provided.** - **We have edited the abstract to remove the inserted headers for “Background”, “ Methods”, “Results”, and “Conclusion” to align with the manuscript style template.** - **We bolded and removed the italics from the figure titles and also de-italicized the figure legends throughout the manuscript per the style template.** - **We bolded table titles and de-italicized table legends throughout the manuscript per the style template.** - **We have added reference to available dataset as supplementary information (line 301), following the example provided in the style template.** |
| Journal Requirement | We note that you have indicated that there are restrictions to data sharing for this study. For studies involving human research participant data or other sensitive data, we encourage authors to share de-identified or anonymized data. However, when data cannot be publicly shared for ethical reasons, we allow authors to make their data sets available upon request. |
| **Response** | **We acknowledge the Journal’s emphasis on ethical data sharing and thank the Journal for working towards an open-data policy. Upon review with our IRB requirements, we are happy to share copies of our de-identified data as Supporting Information files. We have prepared our supplementary dataset following recommendations from BMJ 2010;340:c181. Reference to our data as supporting information is added to lines 301 for the reader. A description of the supplementary dataset is added to the end of our revised manuscript, per the author guidelines.**  **We have updated our Data Availability Statement in the submission form to acknowledge this change.** |
| Journal Requirement | When completing the data availability statement of the submission form, you indicated that you will make your data available on acceptance. We strongly recommend all authors decide on a data sharing plan before acceptance, as the process can be lengthy and hold up publication timelines. Please note that, though access restrictions are acceptable now, your entire data will need to be made freely accessible if your manuscript is accepted for publication. This policy applies to all data except where public deposition would breach compliance with the protocol approved by your research ethics board. If you are unable to adhere to our open data policy, please kindly revise your statement to explain your reasoning and we will seek the editor's input on an exemption. Please be assured that, once you have provided your new statement, the assessment of your exemption will not hold up the peer review process. |
| **Response** | **We acknowledge the Journal’s requirement for accessible data, and we thank the Journal for highlighting the recommendation to review our data sharing plan before publication. In response to the item above regarding data availability, we have made our datasets available as supplementary information. We have updated our data availability statement accordingly.** |

Reviewer Comments:

We thank the reviewers for taking the time to review our initial submission. We acknowledge your comments, critiques, and suggestions. We have made the necessary changes and modifications, where appropriate. Our responses to each reviewer are below.

Reviewer #1:

A general response from the authors (**bold**) to the general comment from reviewer #1: “*Thank you for the opportunity in reviewing the manuscript, it is a well-designed and executed study highlighting novelty within the area of shoulder pain in athletic populations. I have very minor comments, around the consistency of writing and some of the statistical analyses, but it is well written manuscript with very few flaws.”*

**The authors thank the reviewer for their thorough review of our submission and valuable feedback. We have addressed the specific comments in our revised submission and believe that our manuscript is stronger as a result of these changes. Our line-by-line responses to the specific comments from reviewer #1 are included in bold in the table below (please note that line numbers in our responses pertain to the untracked manuscript):**

| Reviewer comment | L21 – Define 3D on first use |
| --- | --- |
| **Response** | **We have modified this line (line 24) to define 3D as “three-dimensional” upon first use in the abstract.** |
| Reviewer comment | L31 – If you state significantly, add the p value to the brackets on line 32. |
| **Response** | **We thank the reviewer for this suggestion. We have added the P-value to this sentence (line 34).** |
| Reviewer comment | L32 – You need a space between 0.8 and mm, there is inconsistency in this though the manuscript. |
| **Response** | **Thank you for catching this oversight. We have inserted a space between the last integer and “mm” at each instance throughout the revised manuscript.** |
| Reviewer comment | L57 - Define 3D on first use |
| **Response** | **We have modified line 61 to define 3D as “three-dimensional” upon first use in the body of the manuscript.** |
| Reviewer comment | L82 – A study is inanimate and cannot compare, please amend accordingly. |
| **Response** | **We appreciate the reviewer’s insight. We agree that the current sentence could be improved. We have amended line 86 to state “Our objective was to compare the 3D…”** |
| Reviewer comment | L88-89 – This sentence seems out of place, consider starting with explaining the experimental design, then this ethics information would be more appropriate. |
| **Response** | **The authors acknowledge the reviewer’s concerns with this section. We have revised lines 92-93 by adding a statement describing the experimental design before the IRB reference, as recommended by the reviewer.** |
| Reviewer comment | L153 – Check the writing style here “(35) (ICC: > 0.91) (36).”, it just needs work. Consider, combining the references so that the ICC is before them both. |
| **Response** | **We thank the reviewer for highlighting their concern with this sentence. We have revised this sentence into two separate sentences to describe, first, the technique and, second, the supporting psychometric information (lines 156-158).** |
| Reviewer comment | L250 – What was the interpretation of the Cohen’s d effect size? Additionally, reporting the 95% CI would also be useful (but not necessary). |
| **Response** | **Interpretation of effect sizes was done according to the description by Cohen in Cohen, J. “Statistical power analysis for the behavioral sciences.” 1988. We have added a description of this interpretation to line 256 and added a citation to Cohen 1988.** |
| Reviewer comment | L256 – Does this create an issue as the majority of the participants are non-swimmers, i.e. non-swimmers vs. swimmers? |
| **Response** | **We thank the reviewer for this question. After successfully matching participants by age and sex for analysis, we further determined that the demographic variables of height, weight, and BMI were not statistically different between groups (swimmers with instability and controls). Therefore, we are confident that the two groups are similar before kinematic analyses.**  **We acknowledge the potential impact of unequal group sizes in a two-factor ANOVA. To address this, we verified that the assumption of homoscedasticity (equal variances) was met, reducing concern for bias related to unbalanced group sizes. Additionally, the limited number of statistically significant differences observed in our analysis suggests a low likelihood of Type I error. Taken together, these findings indicate that any influence of the unequal group sizes on the interpretation of results was likely minimal.**  **In response to a similar comment below (L313 and 391), we have added language to our discussion to acknowledge the data contributed by these two swimmers in the control group.** |
| Reviewer comment | Table 1 – you need to define if the numbers in brackets are the range, could some be the standard deviation? |
| **Response** | **We thank the reviewer for bringing this oversight to our attention. We have revised Table 1 to indicate which variables are represented by either counts (%), mean (range), or mean (∓SD). We have also revised Table 2 to demonstrate the data presented in Table 2 represents counts (%). We believe the two tables are in alignment and clearer for the reader.** |
| Reviewer comment | L313 and 391 – I would like to see some more consideration of the inclusion of only 2 competitive swimmers in the control group, this is potentially a factor that needs observation. Within the results, could you present individual data points where the swimmers could be identified. As you have primarily discussed the p-value, but highlight differences in these 2 control group participants. |
| **Response** | **This is an interesting point and one worth expanding on. Upon reviewing the data from the two swimmers in the control group, it is clear that these two individuals did not stand out in terms of demographics. For instance, their respective BMIs (21.41 & 21.41) are within one standard deviation (3.48) of the mean BMI (23.62). Other demographic characteristics, such as height and weight, follow a similar pattern. Additionally, only one swimmer demonstrated a markedly different ROM profile than the other control participants, with an IR measurement beyond one standard deviation from the control group mean. Language to lines 317-322 has been added to highlight this data.**  **Related to the effect of these two swimmers in the control group on our kinematic results, we have extracted the data of the two swimmers to determine if their data demonstrated trends inconsistent with the rest of the control group. To do so, we evaluated for the presence of outliers in each kinematic analysis (humeral AP, SI, helical axis, and contact path). Overall, one swimmer was detected to contribute outlying data for humeral AP and SI position compared to the control group mean at 30, 45, and 90 degrees of humeral elevation (Note that 30 degrees of elevation was dropped from the analysis due to more than 25% of data being derived from multiple imputation). In our post hoc analysis, removal of this outlying data did not account for any differences in our kinematic comparisons and ultimately confirms our findings that there was only one kinematic difference (avg. AP position) between the two groups. The two swimmers in the control group did not contribute any outlier data to the analysis of humeral IHA or CPL. Additional language to paragraph starting on line 323 has been added to define how the data from these swimmers in the control group impacted our results. Additionally, we have provided figures illustrating the outlying data as supplementary files.** |

Reviewer #2:

A response from the authors (**bold**) to the comment from reviewer #2: “*The results mentioned a significant difference, so why is the conclusion that there is no difference? Perhaps the author could be more detailed in the abstract. Perhaps the author could be more detailed in the abstract.”*

**The authors thank the reviewer for their review of our submission and valuable feedback. We acknowledge the reviewer’s concern related to our conclusion and the abstract. In the abstract, we have added language to lines 36-40 to more clearly state our statistical findings. Our revision to the abstract’s conclusion more closely parallels the conclusion of our manuscript, which more clearly highlights our statistical results. We have added language to our conclusion (lines 438-446) to include our statistical results for the other variables examined (humeral SI position, IHA dispersion, and CPL), thereby clarifying our results for the reader.**
